# Coronavirus Crisis: Government Aid That Also Promotes Employee Ownership

**DOI:** 10.1007/s10272-020-0898-9

**Published:** 2020-06-07

**Authors:** David P. Ellerman, Tej Gonza

**Affiliations:** Faculty of Social Sciences, Uni Ljubljana, Kardeljeva ploščad 5, 1000 Ljubljana, Slovenia

## Abstract

The premise of this paper is that state aid to distressed companies should benefit not only the current owners but also the employees, who are the ones taking personal risks to continue or restart companies. Government aid during the Great Recession was aimed primarily at restoring the status quo. In the current deeper crisis, aid should be designed to create a fairer, more inclusive and more socially responsible economy by promoting employee ownership as both an incentive and a reward. We show how the Employee Stock Ownership Plan, which has been pioneered in the US for 40 years and can be adapted to the European legal context, can be used as the vehicle for structuring this aid.

The Chinese word for 'crisis' combines the characters for 'danger' and 'opportunity'. While the dangers of the ongoing coronavirus crisis are increasingly clear, the opportunities have not been sufficiently explored yet. Our paper focuses on how to utilise the 'opportunity' while saving the economy from the 'danger'.

Around Europe, governments and central banks are currently considering different fiscal and monetary instruments to help liquidity-constrained private and state-owned enterprises. This includes direct government assistance in the form of subsidies, tax breaks, factoring or buying of receivables and forgiving debts as well as indirect aid through national or developmental bank loans.

Governments should ensure that the aid is inclusive and equitable. One option currently being weighed in Europe is nationalisation. A return to state ownership is sensible when it comes to strategic infrastructure or public goods and services such as healthcare and education. However, when talking about more standardised economic goods, and especially when dealing with the structure of ownership in the small and medium-sized enterprise (SME) sector, state ownership is not likely to be desirable.

**David P. Ellerman**, Institute for Economic Democracy, Ljubljana, Slovenia.

**Tej Gonza**, Institute for Economic Democracy, Ljubljana, Slovenia.

An alternative to nationalisation with conventional enterprises is establishing broad-based employee ownership (EO) by using government assistance (Mathieu, 2020; Mitarbeiterbeteiligung, 2020a, 2020b). This paper proposes concrete, practical and bi-partisan policy addenda for governments to use aid packages to establish (part) EO both for private and state-owned enterprises.

The paper suggests that the American Employee Stock Ownership Plan (ESOP) model may be the most relevant example for our purpose and explains how government help can be translated into EO. Subsequently, it outlines the most relevant public policy rationales that support the general case for implementing EO on the EU and individual state level. Finally, the key elements of the proposal are summarised.

## Government interventions and the ESOP model

Different forms of government assistance packages may partly be channelled through dedicated legal entities to establish part EO. We propose that EO be structured according to the American ESOP model. ESOPs are probably the most successful existing practice of EO with around 14 million employees included in the plans, roughly 10% of the private workforce in the US, in several thousand companies of all sizes and sectors (National Center for Employee Ownership [NCEO], 2019).

In a nutshell, ESOP is ownership based on a dedicated trust-like entity associated with a company, which functions as an internal market for shares (and percentages in limited liabilities companies). This trust-like entity maintains ownership among the current employees of a company even through generational transitions of workers.

The ESOP model can be recreated in a European legal context. At the Institute for Economic Democracy, we have designed a model for Slovenia, but the same principles should apply to other European countries. The idea is that the legal entity that would function as an ESOP trust is recognised as a legally constituted cooperative (where a cooperative law applies). The reason for applying the cooperative legal structure is practical, not ideological. As membership institutions, cooperatives are relatively easy to use to organise employee-members with little to no red tape involved. Historical in Europe, cooperatives have for ages offered an alternative way of organising production. We call ESOP cooperatives Co-Op-ESOPs.

## Structuring government aid to establish employee ownership in private enterprises

To understand how this proposal could be part of the financial intervention legislation, we need to first understand its principles. The general principle is when a company receives a grant from public funds, the help (or part of it) should be shared between the existing owners and the employees by making them part owners.

The Slovenian government is considering buying receivables (factoring) from private companies with liquidity difficulties through a special state trust. Let us think first of the underlying idea. A creditor company has a receivable from a debtor company which the debtor company probably cannot pay off. The government comes in through this legislation and buys the receivable from the creditor company, perhaps at a discount. What does the government do with it? If the government 'swallows' the receivable, then it is a wealth transfer to the debtor company whose debt was erased. Hence, debtor companies could be required to agree to the transfer of equal-valued or some portion of shares to the Co-Op-ESOP in return for the government taking the receivable. Is the creditor company also getting a gift? It depends on the actual value of the receivable. If it was really worthless, the price for the purchase of the receivable was also a wealth transfer and some transfer to a Co-Op-ESOP might be appropriate for the creditor company, too. The principles of translating other forms of direct and indirect grants into EO are the same. The general idea is that the value of state help should be matched by newly issued shares or treasury shares, which are transferred to Co-Op-ESOP trusts.

Figure 1

**Government loan channelled through a Co-Op-ESOP**

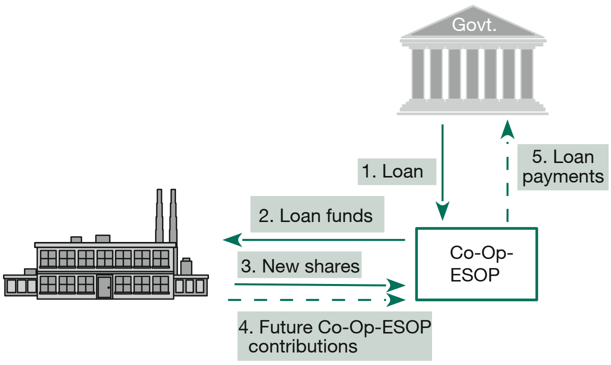

Source: Authors' own illustration.

In the situation where the aid is a liquidity loan rather than an indirect grant (or wealth transfer), the general principle is that part or the whole loan is channelled through a Co-Op-ESOP, which then transfers the money to the company in return for new issues or treasury shares. This shares the ownership by diluting the existing owners and has no cash cost to the company. As the loan is paid off with payments put through the Co-Op-ESOP, the workers get shares equal to their portion of the principal payment into their individual share accounts in the Co-Op-ESOP. The split between workers is usually according to salary. The following explains how a loan would be channelled through Co-Op-ESOP and repaid to developmental or state bank using companies' retained earnings.

In Step 1 (see Figure 1), a government agency makes part (or all) of a loan package through a Co-Op-ESOP that is guaranteed by the Company just like any standard commercial loan. The Company could be either a private company or a state-owned company awaiting future privatisation. In Steps 2 and 3, the Co-Op-ESOP passes the loan funds through to the Company in return for newly issued shares or treasury shares of the Company. In accordance with the loan agreement, the Company will pay back the loan in instalments over a period of years. But as indicated in Step 4, those loan payments will be legally packaged as a contribution to the Co-Op-ESOP (which should be made deductible from taxable income as is the practice followed in the US). The Co-Op-ESOP then passes the loan payments through to the government in Step 5.

The relief loans or subsidies may be made well before a Co-Op-ESOP can be established, but the same net ef-

Figure 2

**Government loan directly to Company with Co-Op-ESOP shares timed with loan payments**

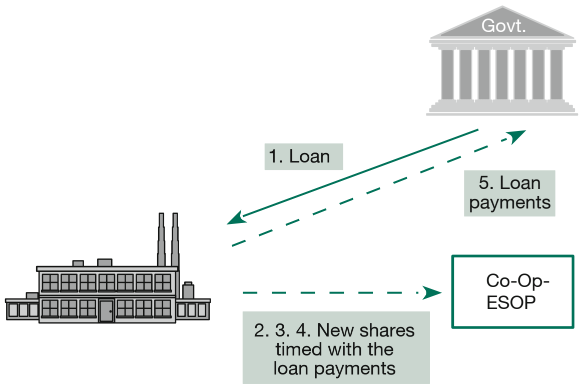

Source: Authors' own illustration.

Figure 3

**Shares allocated to individual share accounts with loan payments**

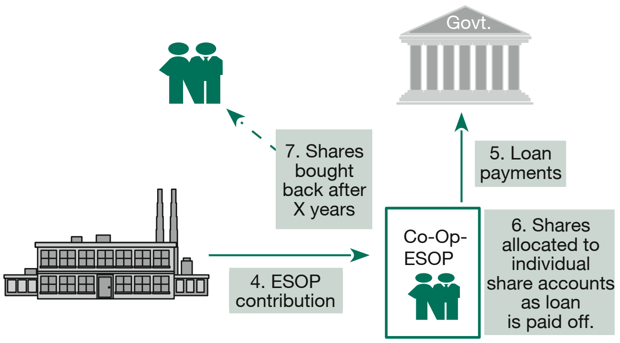

Source: Authors' own illustration.

fects can still be obtained. Then the loan would be made directly to the company but each loan payment would be accompanied by a tax-incentivised contribution of newly issued shares or treasury shares to the later-established Co-Op-ESOP (e.g. with a tax-deductible value equal to the principal portion of the loan payment), as illustrated in Figure 2.

It is only in Step 6 in Figure 3 that shares (equal-valued to the loan principal payment) are allocated to the individual employee share accounts in the Co-Op-ESOP as their reward when loan payments are made. If the Company continues to make Co-Op-ESOP contributions after the loan is paid off, then the Co-Op-ESOP can buy back the employee shares after say X = 5 years (Step 7) and redistribute them to the share accounts of the current employees – to be rolled over again after another X = 5 years.

One important element of this proposal is that there should be a company evaluation for every private company that would have to match the value of government aid with the value of shares transferred to Co-Op-ESOP. For the purpose of this crisis, a universal method of evaluation could be legally determined with which all companies should apply, a method that would not be very resource consuming. For example, the net asset value approach could be used for evaluation.

## Government aid for state-owned enterprises and privatisation

European governments are also putting financial resources into state-owned enterprises (SOEs). The principles of establishing EO in those are the same. When it comes to SOEs using government aid to establish EO, this can be viewed as a partial privatisation in accordance with EU guidelines (where applicable, exempting the companies providing public goods and services that should remain under state ownership).

In Slovenia and other economies where there is a high level of public ownership, many SOEs are in line for privatisation in the upcoming years. Experience shows that impulsive privatisations are often characterised by corruption and underselling. Private investors do not always have the long-term health of the enterprise in mind and may simply want to decapitalise the company.

Introducing part EO to the privatisation model anchors the long-term interest of the company in its employees and the local community. It raises safeguards both when enterprises are owned by the government and when they are owned by a foreign private investor. It also motivates employees in the environment of public employment, infamous for the lack of incentive structures. Finally, EO in state-owned enterprises serves another function if the government decides to sell the enterprises; it is a test for the potential buyer. If the buyer objected to minority EO, it would probably be a reliable signal that the buyer is not interested in the long-term health of the company.

## Doing better than just restoring the status quo

The EU and the member states interested in an EO approach must justify their position on practical as well as policy grounds. EO has proven to be one of the most efficient organisational models for private enterprises. It also enjoys high degrees of social and environmental responsibility. This section outlines some of the most relevant problems addressed by EO.

### Reward and motivation for employees in times of high risk

The first question for the governments providing aid should be who would get the most out of it. Frontline managers and workers in the firms are those taking the personal risks in order to get the economy going again. EO typically increases the wealth (Blasi and Kruse, 2019) and wages of employees (Kardas et al., 1998). Moreover, EO improves workers' quality of life (Bryson, 2016; Erdal, 2014).

### Greater cooperation and co-responsibility

Empirical evidence shows that greater worker autonomy reduces worker-management conflict and leads to greater affiliation for the organisation (Kruse, 2016; Summers and Chillas, 2019). To achieve the full potential in this area, the ownership aspect should be complemented by the change in workplace culture, with the encouragement stemming from a better flow of information, improved management transparency and greater employee involvement and autonomy (Fakhfakh et al., 2012; Kruse et al., 2010, 2008; Perotin, 2016; Rosen et al., 2005).

### Higher resilience and lower employee fluctuation in times of economic crisis

Studies comparing employee-owned companies with conventional companies concluded that the former have 20% to 50% higher survival rates on the markets, with the difference being particularly pronounced in times of crisis (Blair et al., 2000; Blasi et al., 2013; Kruse, 2016).

Kruse et al. (2012) found that workers in the ESOP companies are 50% less likely to voluntarily seek employment in the next year. During the 2007-08 financial crisis, employee-owned companies in the US had between 20% and 50% lower lay-off rates, and it is estimated that during the Great Recession, the US Government saved around $13 billion in unemployment and other programme costs because of EO (NCEO, 2019).

Similar results are found outside the US. In Spain, Mondragon companies were very successful in bridging the 2009 crisis. While the country's unemployment rate rose to 26%, Mondragon collectively decreased wages by 5% to 10% and allocated few redundant members among cooperatives (Tremlett, 2013).

### Superior economic performance of employee-owned enterprises

Employee-owned companies are more successful not only in times of crisis, but also when business is good. Relative to comparable conventional enterprises, employee-owned firms on average enjoy between 4% and 10% higher productivity (Brill, 2012; Freeman, 2007; Blasi et al., 2013; Kruse, 2016), 2.3% greater sales-per-employee growth (NCEO, 2019) and 8.8% faster general growth (Kramer, 2010; Blasi et al., 2017).

### Mitigating the problem of extreme economic inequality

Inequality is a pertinent problem that most governments around the world are trying to address. The European Commission (2018) reports that the current levels of economic inequality are unjust, unsustainable and inefficient – even in one of the most egalitarian economic regions in the world. By democratising the source of income and wealth, the capital, EO addresses this issue without imposing redistributive measures. Recent research from the US shows the average wealth or savings of low- and middle-income employees is $17,000 in conventional companies and $165,000 in employee-owned companies (Blasi and Kruse, 2019).

### Achieving social and environmental responsibility

The current crisis teaches about the importance of local ownership. SMEs are generally considered locally responsible, which has to do with a high degree of community interconnectedness between the owners of local businesses and community members. There is a natural incentive not to foul one's own nest. A sustainable form of EO anchors the local interest with the ownership interest and achieves more socially and environmentally responsible business operations (Denton, 1999; Stranahan and Kelly, 2019; Fifty by Fifty, 2018; Gehman et al., 2019).

### Overcoming the decapitalisation problem

The European Commission, the World Bank and the IMF have, in the past decades, pushed for aggressive privatisation policies in the South and the Southwest Balkans that often resulted in value destruction, wealth expropriation and deepening the economic gap between the workers and corrupt individuals with political connections. Foreign and absentee ownership have their downfalls, and governments should reconsider their privatisation policies.

Overcoming the decapitalisation problem for larger foreign or absentee-owned firms can be addressed by establishing part EO that would guarantee to workers say and oversight, both *de jure* and de facto. Again, employee interest is the interest for long-term sustainability and success of business enterprise, but also for locally responsible operation.

### Arranging ownership succession

Last but not least of the problems addressed by EO is the so-called ownership succession problem for SMEs (Močnik et al., 2019; Duh, 2012; Malinen, 2001; Hnátek, 2013). EO through ESOP trusts is a time-tested mechanism for (gradual) employee buyouts without depending on the employees mortgaging or risking their personal assets. Most of the companies with ESOPs in the US arose out of family succession problems in privately held SMEs, therefore ESOP is often cited as a desirable succession tool in the business literature (Frisch, 2002; Brill, 2017; Flesher, 1994).

## Employee ownership: An opportunity in the coronavirus crisis

The frontline people whose efforts will be needed to save their companies should be supported not simply by their salary but also by the incentive of an ownership reward. This article argues that government aid to companies in need can be channelled so that an inclusive and sustainable model of EO is established.

There is no shortage of reasons why governments and EU should consider EO as an additional goal in addressing the current crisis. It is not difficult to justify government help in establishing EO, since EO is about rewarding employees, who are saving the economy by working in times of crisis. EO is a structural alternative that resides between absentee ownership and economic hyper-globalisation on the one hand, and state ownership on the other. In this respect, it is a 'radical-centrist' idea.

The best model for EO, according to 40 years of experience, seems to be the American ESOP. By explicit design, ESOPs include all the employees in a company. They do not require employees to sacrifice their own savings, and have proven to be sustainable over generations of employees and therefore not simply a windfall for one group of workers. Government grants and loans can be used, at least partially, to establish EO if financial resources (or tax breaks and debt abatements) are channelled through an ESOP-like entity.

The Institute for Economic Democracy,[1] in collaboration with the European Federation for Employee Share Ownership (2020), have developed a generic ESOP model for Europe that, with suitable adjustments, can be implemented in Slovenia or any other European country.

ESOPs build more resilient and efficient enterprises, they anchor capital in local communities and they provide a model for ownership continuation with SMEs. Moreover, ESOPs provide a socially and environmentally responsible economic model. Finally, for state-owned companies awaiting privatisation, EO reduces the state-owned component and serves as a check on buyers not interested in the long-term health of the companies and their surrounding communities.

Instead of resurrecting the established status quo, governmental assistance should use this opportunity to include ordinary employees, whose combined efforts will be required to restore economic health in response to the ongoing pandemic and in years to come.

1 For more information, see https://ekonomska-demokracija.si.
